# Setting Discontinue Rules for Progressive Tests: A Practical and Transparent Toolkit

**DOI:** 10.1177/10731911251385776

**Published:** 2025-11-03

**Authors:** Jianan Chen, Ellen Irén Brinchmann, Johan Braeken

**Affiliations:** 1 University of Oslo, Norway

**Keywords:** discontinue rule, stopping rule, ceiling rule, progressive test, Guttman scale, test design, measurement efficiency

## Abstract

Progressive tests are a popular tailored test format where items are administered in increasing order of difficulty level and discontinued according to a rule system that should counter excessive response burden for test participants and guarantee efficient use of resources for test administrators. To facilitate evidence-based decision-making for setting appropriate discontinue rules, we propose a transparent approach that charts the impact of varied alternative discontinue rules on accuracy and efficiency. These A–E charts are based on retroactively applying discontinue rules to normative item response data. We show that a universal discontinue rule likely does not exist and that the optimal rule varies as a function of the desired efficiency-accuracy trade-off suitable for the intended test use and target population. The proposed approach provides a pragmatic solution for practitioners, researchers, test developers, and test publishers to rethink the existing discontinue rules, systematically evaluate the alternatives, and set appropriate rules.

A popular test design in psychological assessment, for both diagnostic and research purposes, is administering blocks of items in order of increasing difficulty and discontinuing the test when a respondent has made a certain number of errors. This test format, with progressively more difficult items, has been given different names in the literature, such as progressive test (e.g., Raven’s Progressive Matrices; Raven, 1936), cumulative homogeneous test (e.g., Loevinger, 1947), or hierarchical scale (e.g., Sijtsma et al., 2011). Prime examples of progressive tests can be found in the Wechsler intelligence test batteries. For example, eight of the 10 primary subtests in the Wechsler Intelligence Scale for Children—Fifth Edition (WISC-V) test battery feature a progressive structure (Wechsler, 2014). The WISC-V test manual indicates that the Block Design subtest, for instance, consists of 13 ordered items, where subtest administration is discontinued after two consecutive errors of the test participant, and that the Similarities subtest of 23 ordered items is to be discontinued after three consecutive errors.

The design principle underlying these progressive tests is the concept of a Guttman scale (Guttman, 1950). When a perfect Guttman scale holds, that is, all the items measuring a single dimension are ordered strictly from the easiest to the most difficult, a respondent answering correctly on an item is expected to have answered all the preceding easier items correctly; meanwhile, a respondent answering incorrectly on an item is expected to answer all the following more difficult items incorrectly (e.g., Person a to g in Table 1). This enables an intuitive interpretation of the test’s sum score because each score corresponds to a single unique item response pattern (e.g., among Person a to g in Table 1, the only possible item response pattern for a sum score of 3 is 111000). The sum score then precisely defines the ability level, indicating how far an individual reached through the progressive test and hence, indicating which items are beyond their ability and which are within reach. From a validity perspective, these characteristics make a Guttman scale attractive for interpretation, understanding of the construct, and communicating results.

**Table 1.** A Six-Item Guttman Scale: All Seven-Item Response Patterns Perfectly Consistent With the Guttman Principle Versus One Item Response Pattern Illustrating a So-Called Guttman Error.

Person	Item 1	Item 2	Item 3	Item 4	Item 5	Item 6	Score
a	0	0	0	0	0	0	0
b	1	0	0	0	0	0	1
c	1	1	0	0	0	0	2
d	1	1	1	0	0	0	3
e	1	1	1	1	0	0	4
f	1	1	1	1	1	0	5
g	1	1	1	1	1	1	6
h	1	0	1	1	0	0	3

*Note.* Score is the sum score across the six items of the scale. Person h shows a Guttman error on item 2.

The practice of discontinuing the administration of a progressive test, after a number of errors have been made by the participant, can also be brought back to the underlying Guttman design principle. Conceptually, once an individual has reached the “ceiling” of their ability, there is no point in testing further because the outcome on the next more difficult item is already known. A perfect Guttman scale would imply that one can discontinue the test administration immediately after the first incorrect response is observed, because all the subsequent responses to more difficult items will be incorrect. Discontinuing test administration implies that one stops early and that the effective test length is not fixed but tailored to the respondent’s ability level. This is essentially similar to applying a *stopping rule* in a modern computerized adaptive test (CAT; Wainer, 2000) or the rule in the original IQ test by Binet and Simon (1905) to continue testing until one reaches the participant’s *ceiling* ability level.

The main advantage of adaptively deciding to stop test administration is measurement efficiency. An approximately equivalent measure can be obtained with fewer items being administered, saving cost and time for both test participants and administrators. In longer test batteries such as the WISC, total test time can amount to 120 minutes (Wechsler, 2014) in a context where one-to-one individual test administration applies. Reduced test length implies reduced testing time and also less response burden on the participants. For lower-ability participants, going through all the difficult items beyond their ability level would also be increasingly frustrating and demotivating, and would invite random guessing behavior (e.g., Wise, 2017) and test fatigue (e.g., Ackerman & Kanfer, 2009). Hence, one can argue that beyond the obvious benefits for efficiency, the application of a discontinue rule might also lead to more reliable and valid test scores.

While the conceptual logic of a Guttman scale can be ported to the design of a progressive test, a one-to-one literal translation is not possible due to the deterministic nature of a Guttman scale. For a Guttman scale to apply, humans need to behave as flawless item response machines. Yet, even when an item is within reach of a person’s ability, they can occasionally slip up and make an error, and similarly even when the item’s difficulty is beyond reach, a person can make a lucky guess or have some type of specific idiosyncratic prior knowledge or insight that only applies to said item, leading to a correct response instead of the more consistent wrong response. These types of responses, inconsistent with the perfect Guttman response patterns, are also known as Guttman errors (e.g., the deviating item response on Item 2 for Person h in Table 1). In other words, reality dictates that progressive tests are at most probabilistic versions of a Guttman scale. This does not undermine the validity benefits of progressive tests, but makes any statement no longer binary true/false, but true/false in expectation. A person with a sumscore 3 would typically (but not strictly) have the first three items correct and the later items wrong.

Similarly, discontinuing the test as soon as a single error occurs, as prescribed by a strict interpretation of the Guttman principle, is likely also not the best way forward, given realistic human response behavior. A progressive test still facilitates the use of a discontinue rule, but a less strict and empirically supported discontinue rule is desired. A formal discontinue rule needs to be established that defines specific evaluation criteria determining when test administration can safely be discontinued without harming the key measurement properties of the test. If a test is stopped prematurely, measurement accuracy would be negatively affected, as the participant is denied a fair chance to reach their ability level. In contrast, stopping the test too late would bring the before-mentioned risks of response burden, guessing behavior, demotivation, and frustration, and it would make test administration less efficient than it could be.

It is clear that the setting of such a discontinue rule cannot be done through an arbitrary procedure and needs proper justification and empirical support. This is one point where the publicly available documentation of popular progressive tests falls short. When consulting test manuals and reports, we typically find only a brief definition of the suggested discontinue rule without further explanation. WISC-V at least briefly mentions the criteria for their discontinue rules in its technical manual (Wechsler, 2014, p. 49): The rank-order correlation of total raw scores before and after adjustment was at least .98, fewer than 5% of total raw scores changed, and the average raw score difference was at most 2 points. However, it remains unclear why a rank correlation of .98 is “good” or “necessary” or how the 5% or 2 points are linked to the actual efficiency and accuracy of the subtests. The discontinue rules are also not stable from version to version. For instance, the discontinue rule for the fifth version of the Block Design subtest is stricter, allowing fewer errors than in the fourth version. In turn, the fourth version had a more relaxed discontinue rule (i.e., more errors allowed) compared with the third version. In addition, it was based on different criteria; stopping if fewer than 2% of the children passed additional items. This example illustrates that procedures, evaluation criteria, and justifications for setting discontinue rules are not yet well-established nor fully transparent.

While the computerized adaptive testing literature offers procedures for setting discontinue rules (e.g., Babcock & Weiss, 2014; Weiss & Kingsbury, 1984), primarily using Item Response Theory (IRT) model–based methods, the mainstream literature on progressive tests lacks clear general guidelines or default procedures for setting discontinue rules altogether. Hence, as a test user, you could be left in the dark about how a discontinue rule was set for a progressive test and why the discontinue rule was different in different versions of the same test. One may naturally question how these discontinue rules were established and whether alternative better rules exist for your specific measurement purpose and context. Furthermore, the absence of openly accessible documentation does beg the question of what considerations have been made in balancing efficiency and accuracy when setting the discontinue rule for a specific test. This lack of transparency could even raise general concerns about how the choice of discontinue rule affects the validity and fairness of test outcomes.

In this article, we propose a pragmatic toolkit for test developers, practitioners, and researchers to rethink the existing discontinue rules, evaluate alternative rules, and set rules depending on the desired balance between accuracy and efficiency in their measurement context. The toolkit uses charts contrasting measures of accuracy with measures of efficiency across systematically varying discontinue rules that were retroactively applied to normative data (referred to as target A–E charts for short). In what follows, we use a small empirical working example to illustrate the procedure and consider setting discontinue rules under different scenarios using target A–E charts. Practical implications with respect to documentation and reporting standards are discussed, together with directions for future research.

## Evaluating a Discontinue Rule: Measurement Efficiency and Accuracy

As a working example, we consider the Norwegian version of the British Picture Vocabulary Scale—second edition (BPVS-II, Dunn et al., 1997) assessing receptive vocabulary for children aged 3 to 16. This test is used, for instance, in the assessment of language impairment or cognitive developmental research. The BPVS-II is a progressive test that consists of 144 items in total that are administered in 12 item blocks with increasing block difficulty, each consisting of 12 items with similar item difficulty. The test follows a one-to-one test administration format, where for each item, the test-taker is asked to choose the picture corresponding to the stimulus word provided by the test administrator from among four alternatives. The current data is part of a larger longitudinal study (Brinchmann et al., 2019) where n=209 six-year-old children (mean age = 6.3 years, age range = 5.8–6.8 years, approximately 50% were girls) were administered this progressive test. The discontinue rule states that the test administration is discontinued after a block in which the child made eight or more errors.

An ideal discontinue rule needs to be maximally efficient in the sense that it reduces test time or test length (i.e., number of items administered) for most participants, while not sacrificing accuracy (e.g., resulting individual test scores hardly change compared with the original scores). To then assess the accuracy and efficiency under a specific candidate discontinue rule, a reference baseline for the chosen evaluation criteria is needed and a typical choice for this in the context of a progressive test, is to compare to results without the use of the discontinue rule, or in other words, under a full test administration (i.e., all items administered for every individual).

In the absence of item response data under full test administration, we cannot fully assess the quality of the existing default discontinue rule, as we would not know what each individual participant would have responded to the remaining unadministered items. However, what can be done is to evaluate a stricter discontinue rule than the current default, where the test would, for instance, be discontinued after four errors instead of the default eight errors. The item response pattern of each individual is then rescored by retroactively applying the new candidate discontinue rule: each item response (block) after four errors within a block is now considered unadministered instead of observed. All evaluation criteria for accuracy and efficiency are then recomputed based on the rescored item response data, and compared with those for the original data under the default discontinue rule.

### Measurement Efficiency

Consider a progressive test that consists of J items in total. Under a discontinue rule, not all items are necessarily administered and test length will potentially vary by person. We will denote the resulting person-specific test length as $J_{p}$ . To distinguish between test lengths under different discontinue rules, we use $J_{p}\left(4\right)$ for the test length under the candidate discontinue rule of four errors within a block and $J_{p}\left(8\right)$ for the test length under the default discontinue rule of eight errors.

Logically, the stricter a discontinue rule is (i.e., allowing test discontinuation with fewer errors), the faster a person’s test will likely be stopped and the fewer items are administered, resulting in shorter test length. For our BPVS working example, when comparing the candidate discontinue rule of four errors within a block to the default discontinue rule, the median test length would be reduced by 33% (i.e., 36 items), decreasing from 108 items to 72 items, and lead to a more homogeneous test length across individuals in the sample, with test lengths ranging in the interval $J_{p}\left(4\right)=\left[48,84\right]$ instead of the original range of $J_{p}\left(8\right)=\left[60,144\right]$ (see Figure 1A). Whereas under the default discontinue rule, five individuals (2% of the sample) had to complete the full test until the last item (Item 144), the candidate discontinue rule would lead to an efficiency gain of at least two full blocks of 24 items for 84% of the sample and everyone’s test was now discontinued before reaching the end of the progressive test (see Figure 1B). Depending on the test context and purpose, different operationalizations of measurement efficiency can be chosen as evaluation criteria: e.g., median test length, the percentage of individuals for whom the test administration was not discontinued, or the percentage of individuals with a given minimum reduction of items administered (testing time).

**Figure 1.**
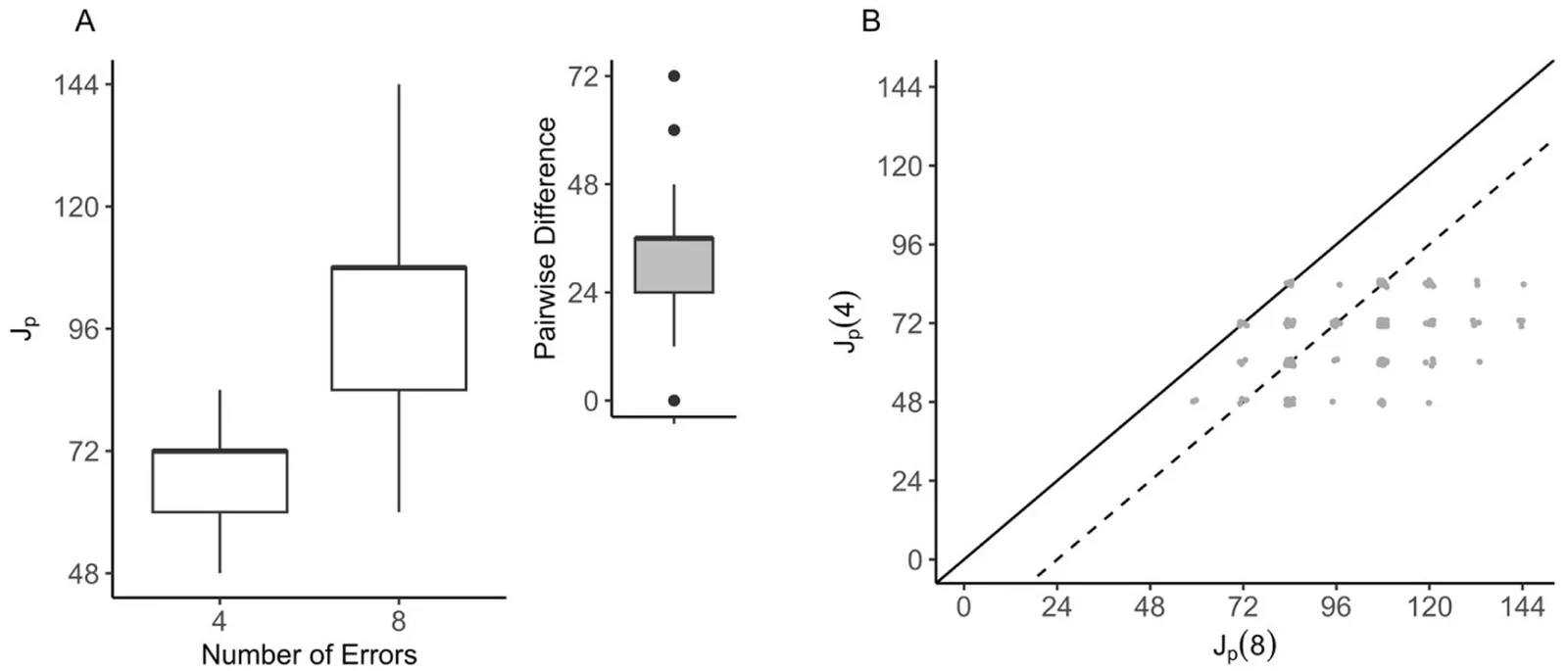
Measurement Efficiency of Stopping After Four Errors Versus Eight Errors Within a Block in BPVS. *Note.* In Panel A, $J_{p}$ represents the person-specific test length. The gray boxplot summarizes the distribution of the pairwise difference between the two discontinue rules in person-specific test length. In Panel B, each gray dot represents an individual, with $J_{p}\left(4\right)$ the person-specific test length under the candidate rule of stopping after four or more errors within a block and $J_{p}\left(8\right)$ the person-specific test length under the default rule of stopping after eight or more errors within a block. Dots on the solid diagonal line of correspondence have equal test lengths under the two discontinue rules. Dots on or to the right of the dashed diagonal line have a test length reduction of at least two blocks (24 items) in the four-error discontinue rule compared with the eight-error discontinue rule.

### Measurement Accuracy

The most apparent impact of applying a discontinue rule is the potential change observed in the score on the progressive test for a specific individual (e.g., Ferman et al., 1998). The sum score $Y_{p+}$ for a person p across all items on the test is commonly used for individual measurement purposes (Sijtsma et al., 2024). Under a discontinue rule, not all items are necessarily administered and we will denote the corresponding test score as $Y_{p+}^{\prime }=\sum_{j=1}^{J_{p}}Y_{pj}$ , where the sum is taken across all items administered up to the point that the discontinue rule indicates that the test should be stopped. A discontinue rule leads to a test score that is lower than or equal to the test score under full test administration (i.e., $Y_{p+}^{\prime }\leq Y_{p+}$ ), because after discontinuation of the test, all remaining test items can no longer contribute to the score. An equivalent statement holds when comparing a stricter discontinue rule to a more relaxed discontinue rule. In our BPVS working example, when contrasting the test scores when discontinuing after four or eight errors, the median and maximum test score differences across individuals were substantial, at 15 and 39 points, respectively. In practice, such large score differences with the default discontinue rule would imply that accuracy under the new candidate rule is rather questionable. Depending on the text context and purpose, different operationalizations of measurement accuracy can be chosen as evaluation criteria: e.g., the percentage of individuals with unchanged scores, the rank-order correlation between test scores under the alternative discontinue rule and the baseline, the change in group-level descriptive statistics, or the change in classification outcomes.

## Mapping the Accuracy-Efficiency Trade-Off Across Discontinue Rules

While choosing the evaluation criteria of interest in terms of efficiency and accuracy is one of the key decisions to take when considering a new discontinue rule, there are several considerations to make when searching for an optimally suited stopping rule given a specific testing context.

First, the structure of the candidate discontinue rules needs to be formulated. A discontinue rule based on the number of consecutive errors is generally suitable for progressive tests because it reflects an item-level Guttman pattern aligning closely with the progression of item difficulty from item to item. However, there may be exceptions, especially for tests administered with blocks of items. An example is the second version of the Test for Reception of Grammar (TROG-2; Bishop, 2003). This test consists of 20 item blocks that increase in difficulty and each contains four items that measure a single grammatical rule using different stimuli. While item blocks are ordered, items within a block are considered equally difficult. In the administration of TROG-2, the decision was made to define the discontinue rule at the block level instead of the item level and the test is discontinued after five consecutively failed blocks, where a block is considered failed if any of its four items are answered incorrectly. A failed block is considered an indication that the grammatical rule is not fully mastered. Our BPVS working example has a similar test structure with 12 ordered blocks, each with 12 items of similar difficulty within the block. Here, an alternative discontinue rule structure is chosen, where the test is discontinued if eight or more errors are made within a block.

The choice between these three variants, (a) stopping after *X* consecutive failed items, (b) stopping after *X* consecutive failed blocks, or (c) stopping after reaching an error rate *X* within a block, depends on the conceptualization of mastery of aspects of the to-be-measured construct and the corresponding test design. When the target construct measure is conceptualized as more of a clear continuum without distinct categorical aspects (e.g., the Picture Span subtest in WISC-V, where working memory is assessed through the task of recalling sequences of pictures that increase in sequence length), a progressive test design without blocks and a corresponding discontinue rule in terms of consecutive failed items is a logical choice. When the target construct measure is conceptualized as more of a level-graded continuum (e.g., vocabulary as in BPVS), a progressive test design with blocks and a corresponding discontinue rule in terms of reaching a given error rate within a block would be the better match. When the target construct measure is conceptualized as a level-graded scale with distinct categorical aspects (e.g., grammar rules as in TROG-2), a progressive test design with blocks and a corresponding discontinue rule in terms of consecutive failed blocks is then the logical match. Of course, these are mere guidelines, but the key point is to design a new discontinue rule in line with the content, structure, and purpose of the specific progressive test.

Once the structure of the discontinue rule is set, a choice in evaluation criteria needs to be made that allows finding a good balance between gaining efficiency while retaining accuracy. Both the evaluation criteria and what balance to aim for depend on the test purpose and context of test administration. While it is impossible to provide guidelines for all possible use cases, we will illustrate the main principles under a few typical scenarios where the focus is either on (a) individual measurement in a high-stakes testing situation, (b) assessment of individual differences for research purposes, or (c) preliminary screening for further follow-up.

With the discontinue rule structure and evaluation criteria in hand, the next step is to compare candidate discontinue rules of different strictness in terms of X the number of errors allowed. This can be achieved by retroactively applying these discontinue rules to empirical data and mapping these rules in terms of the reached efficiency and accuracy. Figure 2 illustrates such a chart where test length in terms of the median number of items administered, is plotted on the horizontal axis as an efficiency evaluation criterion versus an accuracy evaluation criterion on the vertical axis, such as the maximum individual score difference in the sample. Each dot in the plot represents a discontinue rule that stops after *X* number of errors within a block, with the value for *X* provided as an annotation next to the dot. The resulting chart shows a monotonically nondecreasing trend, accuracy remains stable or decreases as the discontinue rule becomes stricter (*X* decreases), while the median number of items administered either remains stable or decreases. We will refer to this type of visualization as a *target A–E chart*. Such a visualization of the evaluation criteria as a function of a realistic range of possible discontinue rules provides an intuitive way to make informed decisions on the optimal candidate discontinue rule.

**Figure 2.**
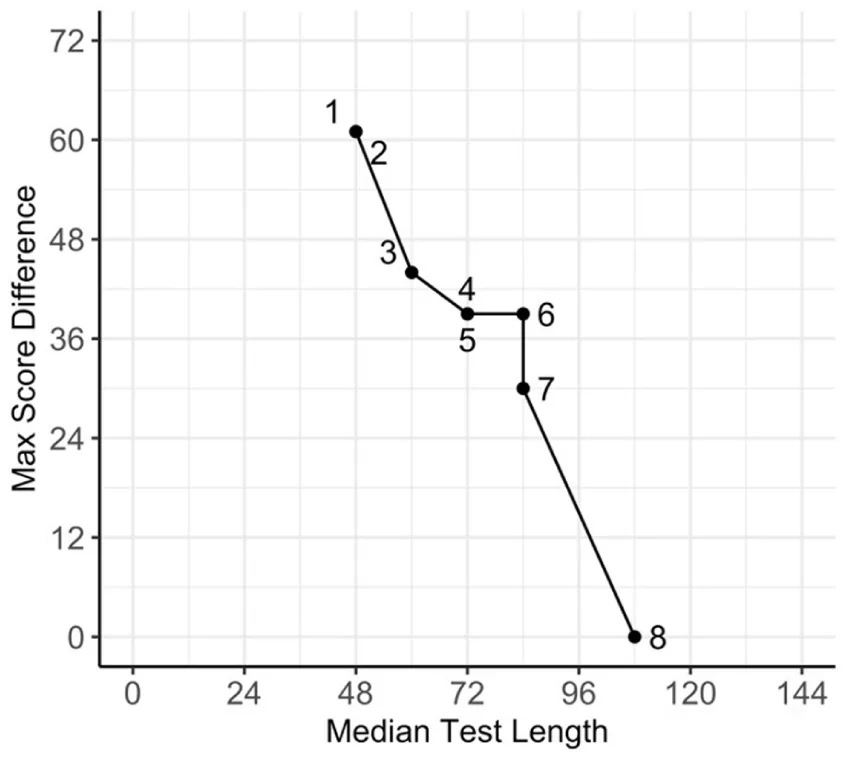
High-Stakes Scenario: A–E Charts of Stopping After X or More Errors Within a Block in BPVS. *Note.* Each dot represents a single discontinue rule of *X* or more errors (incorrect responses) within a block. The value of *X* is indicated by the number-label next to each dot. X=8 is the current default discontinue rule.

Custom functions have been written in the free software environment for statistical computing and graphics R (R Core Team, 2024) to retroactively rescore a sample dataset of item responses given a redefined discontinue rule and to construct A–E charts for specified evaluation criteria. The code and data behind the analyses and figures in this manuscript have been made publicly available at the Open Science Framework: https://osf.io/58bqr/?view_only=a0ba40df1df644319689fabfda5edebd.

### Individual Measurement in a High-Stakes Testing Situation

In high-stakes testing situations where the test has major consequences on the individual respondents, such as in diagnostic assessments, the highest possible accuracy should be prioritized to ensure the fairness and validity of the test outcomes. The primary objective of choosing a discontinue rule in such scenarios is to minimize the differences in individual scores. If the goal is to achieve 100% accuracy in individual test scores, the optimal discontinue rule is the most efficient rule that allows for a maximum score difference of zero (i.e., no change compared with the original score). For our BPVS working example, we can only rely on the default discontinue rule of X=8 errors within a block as a reference point instead of complete administration of the progressive test. Still, any alternative discontinue rule should not do worse than the current default.

If we prioritize efficiency a little bit more, and stop after seven instead of eight errors in the same block, the median test length would be reduced by about 24 items from 108 to 84 items. Yet, when X=7 , only one less error than the default discontinue rule would dictate, an individual’s sum score could be biased downward by as much as 30 points (corresponding to a 58 drop in percentile rank) compared with the original rule (see Figure 2). In other words, for a specific individual, the implications of choosing a specific discontinue rule could be dramatic. This further stresses the need for a strong justification and a transparent empirical basis when setting a discontinue rule for a progressive test.

### Assessment of Individual Differences for Research Purposes

Outside of clinical or diagnostic use, progressive tests are also widely used for research purposes to assess individual differences in constructs of interest. This shifts the primary focus from a single individual case to a group of individuals varying in performance. This shift in focus would also be logically reflected in setting a discontinue rule for research purposes. For our working example, one can argue for the use of a discontinue rule that discontinues after seven errors within a block instead of eight. This adjustment would bring back the median test length from 108 to 84 while preserving the scores for 60% of the group, resulting in a rank-order correlation of .88 between scores under the two discontinue rules (see Figure 3) and only limited differences in the central tendency and variability of performance in the group ( $M^{\left(8\right)}=73,\;SD^{\left(8\right)}=11.9$ versus $M^{\left(7\right)}=69,\;SD^{\left(7\right)}=11.8$ ).

**Figure 3:**
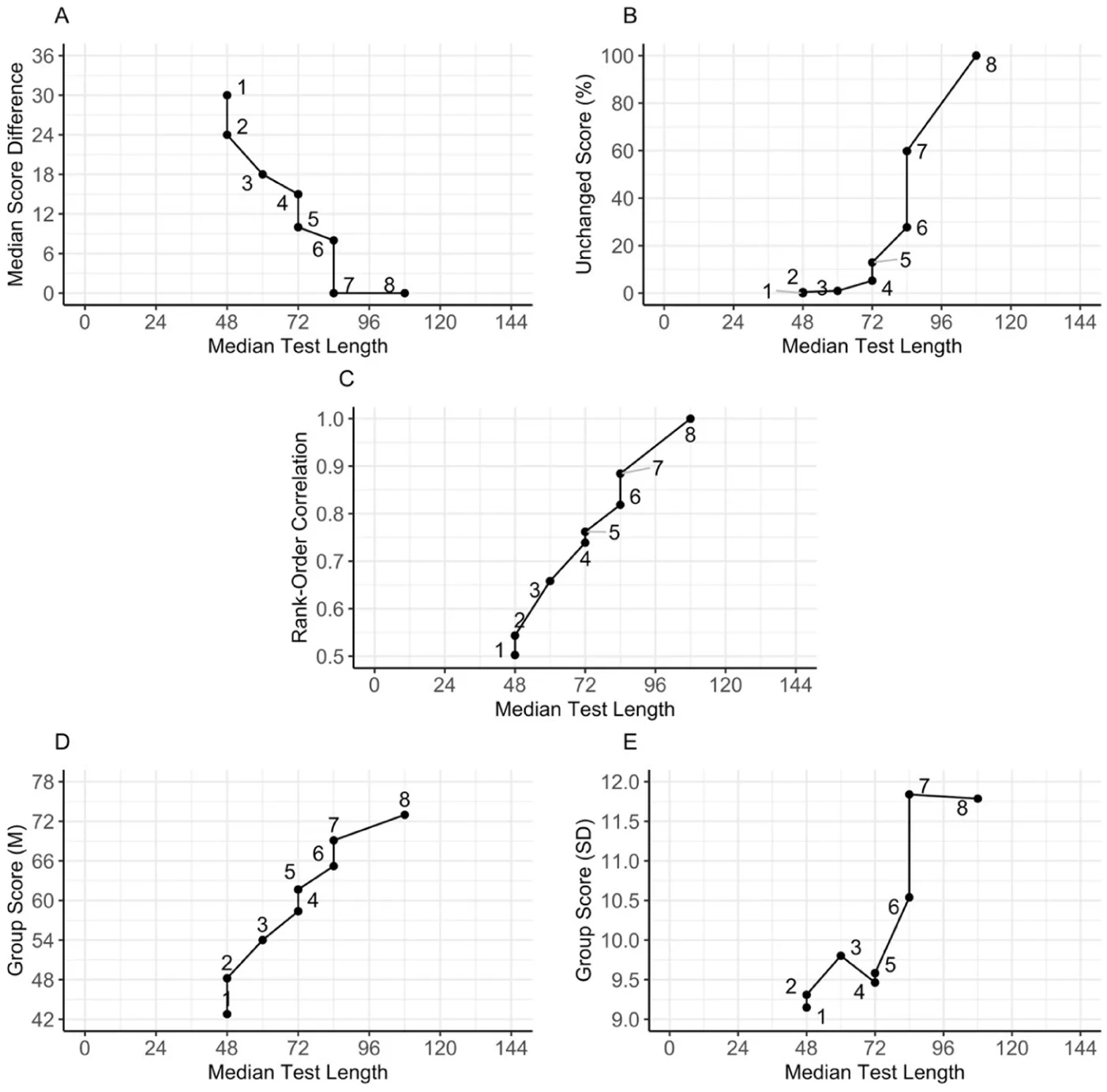
Research Scenario: A–E Charts of Stopping After X or More Errors Within a Block in BPVS. *Note.* Each dot represents a single discontinue rule of *X* or more errors (incorrect responses) within a block. The value of *X* is indicated by the number-label next to each dot. X=8 is the current default discontinue rule.

Prioritizing efficiency further by adopting a discontinue rule of five errors within a block would reduce the median test length to 72 items. However, this comes at the cost of accepting a median score difference of 10, with the group’s average score dropping from 73 to 62 and the group’s standard deviation from 11.8 to 9.6. The latter changes in the summary statistics of the resulting test score might be substantially different from the original. The rank-order correlation of the scores under the five-error and the default eight-error discontinue rule was .76 (Figure 3C), which might raise concerns about the reliability of the statistical inferences drawn from a research study using the five-error discontinue rule. Hence, the latter discontinue rule can be considered too liberal for practical use.

### Preliminary Screening for Further Follow-Up

Efficiency should be prioritized in some scenarios where time and resource constraints are more important than perfect accuracy. One example is when a test is used for large-scale preliminary screening. The exact score of an individual matters less than the more categorical observation that their score is above or below a certain threshold. Hence, less stringent constraints can be put forward for accuracy than for individual measurement (where an individual’s exact position matters and not merely the category they belong to), and a more natural evaluation criterion than score differences would be classification differences.

For instance, in Norwegian schools, national screening tests for reading compare each Student’s test performance to a threshold score corresponding to approximately the 20th percentile in the population. Students scoring below this threshold score are followed up by the teacher or a local school psychologist. Ideally, we would like to make the same decision for each individual regardless of whether the discontinue rule was applied. Yet, under a stricter discontinue rule, we risk having more individuals held back for follow-up due to the potentially lower test scores. In misclassification terminology, this type of error is called a false positive. Although the consequence of a false positive in this context can be regarded as not severe for the child (if the context does not imply a negative labeling bias), the increased costs for follow-up of many more children could potentially strain the school system and the successful implementation of the follow-up.

Figure 4 shows the percentage of false positives^1^ against median test length, and let us first focus on the 6-year-old sample, displayed at the far right. When tightening the discontinue rule from eight to seven errors, the percentage of false positives goes up by only 5%, yet with a median reduction in test length of 24 items. To further reduce the median test length by one third (from 108 to 72 items), a discontinue rule of five errors within a block could be adopted, resulting in 24% false positives. While halving the test length is possible by reducing the number of errors in the discontinue rule from eight to two, 75% false positives would likely not be an acceptable outcome for the screening procedure. In other words, it is advisable to conduct a cost-benefit assessment of false positives for the screening procedure in advance and then set an a priori acceptable rate of false positives. This is a bit similar to deciding on an acceptable significance level or doing a power study in statistical inference.

**Figure 4.**
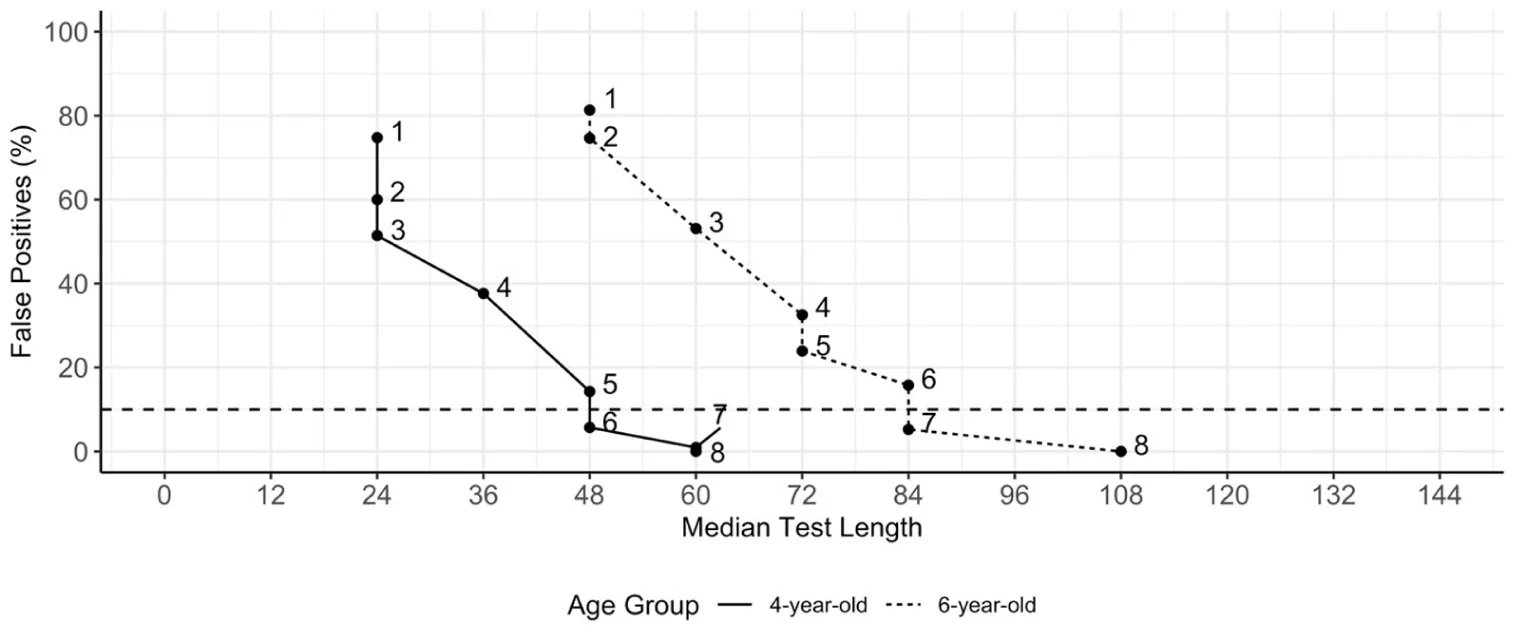
Screening Scenario: A–E Chart of Stopping After X or More Errors Within a Block in BPVS for two Age Groups. *Note.* Each dot represents a single discontinue rule of *X* or more errors (incorrect responses) within a block. The value of *X* is indicated by the number-label next to each dot. The larger the number of errors allowed, the more relaxed the discontinue rule (i.e., it stops conservatively). X=8 is the current default discontinue rule. The horizontal dashed line indicates a threshold of 10% of false positives.

#### Age-Dependent Discontinue Rules

In addition to test purposes, target populations may also influence the choice of the optimal discontinue rule. For most progressive tests used in common practice, the manual states a single discontinue rule based on normative data from a population that is quite broad in age range. Given that norms tend to be a function of age, one might also question whether a single discontinue rule is optimal for use across all age groups. For our BPVS working example, we also had access to empirical data from the participants at a younger age (4 instead of 6 years old), which was part of the longitudinal study (Brinchmann et al., 2019). Figure 4 compares the impact of discontinue rules on the percentage of false positives against median test length for 4-year-old and 6-year-old samples. Compared with older children, the number of items administered to younger children is already lower under the default discontinue rule, but additional efficiency gains are possible without significantly compromising accuracy. For instance, in the screening low-performers scenario, if a false positive rate below 10% is required, the charts suggest stopping after seven errors for the 6-year-olds, but after only six errors for the 4-year-olds (Figure 4). While the change in the discontinue rule may not seem large, the actual reduction in test burden can be substantial.

## Discussion

In mainstream test review models for assessing the quality of tests, discontinue rules are not listed as a point of attention. For example, neither the (American) Standards for Educational and Psychological Testing (AERA et al., 2014) nor the European Federation of Psychologists’ Associations Test Review Model (EFPA, 2013) includes any guidelines or standards regarding how discontinue rules are established or documented. It is perhaps then no surprise that, in practice, the development procedures for the discontinue rule are often undocumented and lack clear justification in publicly available test manuals. Yet, the above scenarios illustrated that there are many considerations to make when setting a discontinue rule and that the choice for a specific discontinue rule might vary depending on the intended test purpose, test context, target population, evaluation criteria, and desired balance between measurement efficiency and accuracy. The implications of choosing a specific discontinue rule can potentially be dramatic for individual scores, but can also impact norm tables, screening decisions, and other test inferences. Hence, having an adequate discontinue rule is an essential aspect of the valid use of a progressive test. Altogether, this stresses the need for a stronger justification and transparent empirical basis for a discontinue rule in a progressive test. We hope that the current manuscript increases awareness around this issue among test users, test developers, and test publishers. By bringing the validity aspect of discontinue rules out of the shadows, it also opens up the opportunity for further updating and upgrading available test review and documentation standards.

Most progressive tests in psychological assessment default to a single discontinue rule that is assumed to be universally applicable and optimal, regardless of, for instance, test purpose or target population. The above scenarios illustrated that this universality assumption of discontinue rules might be questionable and that we might want to have the opportunity to adapt a discontinue rule when the testing context asks for this. In practice, norm tables are already developed and used as a function of the age of the participants, and also the starting rule of a progressive test is typically set as a function of age (i.e., too easy items are not administered and considered correct from a certain age, unless counter-indications arise). For clinicians assessing young, vulnerable children, being able to cut back on testing time without compromising accuracy would be important. For researchers, being able to measure and do more within the limited time and resources available would also be extremely attractive. Therefore, the need for flexibility in adjusting discontinue rules should receive attention.

The proposed toolkit to retroactively evaluate discontinue rules through the inspection of Accuracy-Efficiency charts could serve as a pragmatic and transparent solution for stepping away from the arguably unrealistic notion of a universally applicable default discontinue rule, and instead move toward setting evidence-based discontinue rules targeted to specific scenarios. The approach would require that publishers of progressive tests provide discontinue rule A–E charts of relevant evaluation criteria as illustrated throughout the manuscript with our BPVS working example. These charts would be preferably based on normative data acquired under full test administration. However, even when response data were collected under a default discontinue rule, comparisons with stricter discontinue rules remain feasible. Having such A–E charts available, preferably in the form of an interactive web-based dashboard, allows for full transparency in the empirical basis of the current default discontinue rule, and also provides practitioners and researchers with flexibility in considering alternative discontinue rules, evaluating their potential implications, and choosing the rule that best aligns with their specific testing purpose while achieving the desired balance between efficiency and accuracy. The fact that the final decision is human-made, based on priorities and cost-benefits assessed for the test purpose at hand, is something we see as an advantage. The approach remains feasible to implement in practice, does not require specialized expertise of complex black-box machinery common in more measurement-model-based approaches, and therefore allows for clear, intuitive communication of how decisions were made. It does place a certain responsibility on the end-user, which not everyone might be comfortable with, due to the lack of a straightforward rule of thumb (cf. discussion on justifying the sample size for a study, Lakens, 2022). Thus, it is advisable to still provide a suggested default conservative discontinue rule in the documentation for a specific progressive test.

While a single universal rule facilitates learning the test administration procedure (e.g., WISC-V sets the same discontinue rules across subtests to ease memorization for test administrators; see Wechsler, 2014, p. 31), having more options in test administration of a single progressive test might require more intensive training and instruction in the specific test at hand for new test administrators. On the other hand, recent experimenting with digital or even remote administration of, for instance, the WISC (e.g., Wright, 2020) indicates that computer-assisted administration of progressive tests might be a feasible and realistic feature. While technological developments can help manage test administration complexities, they cannot take away the essential informed decision-making process in setting the appropriate priorities when choosing which variant of the discontinue rule to apply in a progressive test.

When comparing test scores across administrations that have applied different discontinue rules, a question that can arise is whether the same construct is being measured in each case. This would be most relevant when the discontinue rule is explicitly communicated to the participant prior to testing. A stricter discontinue rule then implies communicating higher demands, the expectation that the participant needs to perform more consistently on the test, and this could potentially influence test performance through a range of psychological factors (e.g., motivation or stress) that are not necessarily construct-relevant (AERA et al., 2014; Cronbach & Meehl, 1955). Without explicit communication, such construct-irrelevant variance issues are less prominent. However, the implied differences in accuracy between the test administrations are still a factor in the comparison that one needs to remain aware of.

An important aspect of the validity of a progressive test is that its assumed item difficulty ordering indeed applies to the group being tested. Ideally, in tests without item blocks, item difficulty should increase strictly from one item to the next. In tests administered with item blocks, items within each block are assumed to be of equivalent difficulty, with difficulty increasing strictly from one block to the next. If the item difficulty order does not hold consistently across different groups of test-takers, the effectiveness of a discontinue rule will also highly vary across those groups. In psychometrics, this assumption is called invariant item ordering (see, for example, Koopman & Braeken, 2025), and a violation of it would be an instance of strong differential item functioning, where item bias against a specific group distorts the assumed item ordering. For instance, in performance tests for language, such as assessments of grammar or vocabulary, the observed empirical item difficulty (e.g., item proportion correct) typically aligns with the normative age of acquisition of the specific grammatical rule or word central to the item in the target population. Yet, several environmental and cultural factors can affect this age of acquisition such that specific groups do not align well with what is typical for the normative target population. Administering a test with an invalid item order for the group being tested will naturally lead to the default discontinue rule, based on the item order, being overly strict. This is because we can no longer rely on the fact that once the first sequence of incorrect responses (i.e., errors) is observed, the subsequent items will also be answered incorrectly. In other words, the underlying Guttman design principle of the progressive test would no longer apply, breaking the logic underlying the discontinue rule. In contrast, the stronger the item order in the progressive test, the more effective the application of a discontinue rule can be. Hence, while setting a transparent and justifiable discontinue rule is important, it is only one aspect of developing a progressive test and answering the broader question of test-use validity (see, for example, Kane, 2013). The efficiency gains of discontinue rules can be fully realized only when a test is optimally designed to support their use. However, even when invariant item ordering does not hold, or when an ideal item difficulty structure is not feasible (e.g., when items within a block cannot be of the same difficulty), our proposed approach remains useful for setting optimal discontinue rules. By using empirical response data, the A–E charts are based on observed responses to the actual test items, thereby capturing the impact of less-than-ideal design features.

Another factor that will determine how strict of a stopping rule can be set, is the prevalence of guessing behavior. This is especially relevant in progressive tests that use multiple-choice formats, where respondents might answer items correctly by chance without truly mastering the construct, or answer items incorrectly simply due to disengagement (e.g., Wise, 2017). The more guessing behavior occurs in the population, the more relaxed the discontinue rule needs to be to accommodate these deviations from the ideal Guttman response pattern. Eliminating guessing behavior in practice is challenging and the occurrence of guessing behavior might depend not only on gender and cultural differences, but also on test instructions and allowed time for response. Again, a universal stopping rule for a test likely does not exist and depends on the context and purpose the test is used for. The proposed A–E charts will tune in on the empirical characteristics of the sample data used to set the stopping rule, but the efficacy of the rule will necessarily depend on the match between the normative sample and the intended test use, context, and target population.

One aspect not explicitly addressed in the current study is incorporating measurement error in the scores while setting the stopping rule. Quantifying measurement error would require adopting a measurement model for the tests of different lengths (e.g., classical test theory) or an IRT model for the full item bank. While not commonly done in traditional progressive testing, one could consider stopping criteria similar to operations in computerized adaptive testing (CAT; Wainer, 2000) in terms of the attainment of a predefined threshold of standard error of measurement or its convergence around the latent trait estimate. While the application of the CAT-based approach in individual clinical testing might encounter resistance due to perceived complexity and limited transparency, the ongoing advancements in accessibility of information and communication technology might still make it a potentially promising direction for further research.

## Conclusion

Although discontinue rules are widely used in administering progressive tests in psychological assessment and enhance both efficiency and validity, two primary concerns need addressing: (1) The decision-making process in setting discontinue rules is often missing or inadequately documented and justified. (2) Default discontinue rules provided in test manuals are considered universally optimal, without consideration of the test’s purpose or use-context. To address these concerns, we proposed a pragmatic solution using target A–E charts that contrast efficiency with accuracy for relevant criteria across a range of systematically varied discontinue rules applied retroactively to normative data. We call for more attention to the implications of using different discontinue rules and more transparent and context-specific decisions on setting discontinue rules for progressive tests.

## Data Availability

The code and data behind the analyses and figures in this manuscript have been made publicly available under a CC BY-NC-SA license at the Open Science Framework: https://osf.io/58bqr/?view_only=a0ba40df1df644319689fabfda5edebd.

## Notes

1. In different contexts, false negatives or the total percentage misclassified or any other summary statistic of the confusion matrix could be relevant.
